# Antimicrobial activity of Mycobacteriophage D29 Lysin B during *Mycobacterium ulcerans* infection

**DOI:** 10.1371/journal.pntd.0007113

**Published:** 2019-08-19

**Authors:** Alexandra G. Fraga, Gabriela Trigo, Ramya K. Murthy, Shamim Akhtar, Madhavi Hebbur, Ana Rita Pacheco, Juan Dominguez, Rita Silva-Gomes, Carine M. Gonçalves, Hugo Oliveira, António G. Castro, Umender Sharma, Joana Azeredo, Jorge Pedrosa

**Affiliations:** 1 Life and Health Sciences Research Institute (ICVS), School of Health Sciences, University of Minho, Braga, Portugal; 2 ICVS/3B’s—PT Government Associate Laboratory, Braga/Guimarães, Portugal; 3 Centre of Biological Engineering, University of Minho, Braga, Portugal; 4 GangaGen Biotechnologies Pvt Ltd., Bangalore, India; Stanford University, UNITED STATES

## Abstract

Buruli Ulcer (BU) is a cutaneous disease caused by *Mycobacterium ulcerans*. The pathogenesis of this disease is closely related to the secretion of the toxin mycolactone that induces extensive destruction of the skin and soft tissues. Currently, there are no effective measures to prevent the disease and, despite availability of antibiotherapy and surgical treatments, these therapeutic options are often associated with severe side effects. Therefore, it is important to develop alternative strategies for the treatment of BU. Endolysins (lysins) are phage encoded enzymes that degrade peptidoglycan of bacterial cell walls. Over the past years, lysins have been emerging as alternative antimicrobial agents against bacterial infections. However, mycobacteria have an unusual outer membrane composed of mycolylarabinogalactan-peptidoglycan. To overcome this complex barrier, some mycobacteriophages encode a lipolytic enzyme, Lysin B (LysB). In this study, we demonstrate for the first time that recombinant LysB displays lytic activity against *M*. *ulcerans* isolates. Moreover, using a mouse model of *M*. *ulcerans* footpad infection, we show that subcutaneous treatment with LysB prevented further bacterial proliferation, associated with IFN-γ and TNF production in the draining lymph node. These findings highlight the potential use of lysins as a novel therapeutic approach against this neglected tropical disease.

## Author summary

Buruli Ulcer (BU) is a necrotizing skin disease caused by *Mycobacterium ulcerans*. Although the current antibiotic treatment for BU is effective, daily administrations for a prolonged period of time, combined with potential risk of severe side effects, negatively impact on patient adherence. In that sense, we tested the efficacy of an alternative strategy based on Lysin B (LysB), a phage encoded lipolytic enzyme that degrades the mycolylarabinogalactan-peptidoglycan complex present in the mycobacterial cell wall. In this study, we show that LysB not only displays lytic activity against *M*. *ulcerans* isolates *in vitro*, but also leads to a decrease of *M*. *ulcerans* proliferation in infected mouse footpads. These findings highlight the potential use of lysins as a novel therapeutic approach against this neglected tropical disease.

## Introduction

Buruli ulcer (BU) is a necrotic cutaneous disease caused by *Mycobacterium ulcerans* and represents the third most prevalent mycobacterial infection worldwide, after tuberculosis and leprosy [1].

BU pathogenesis is closely related to the secretion of the polyketide toxin mycolactone that presents cytotoxic and immunosuppressive properties [2–4]. Early presentations of active BU include a painless pre-ulcerative nodule, papule, plaque or edematous lesion, which can evolve into typical ulcers or, in the most extreme cases, may result in extensive skin destruction, multifocal lesions or bone involvement [5].

The standard antibiotic regimen recommended by the World Health Organization (WHO) consists of daily administration of rifampicin and either clarithromycin or streptomycin for a period of 8 weeks [6,7]. Despite its proven clinical efficacy, the prolonged administration associated with potentially severe nephrotoxic, hepatotoxic and ototoxic side effects [6] and the possibility of the emergence of drug resistant strains [8], render the search for alternative treatments a necessity.

Our group has previously demonstrated that bacteriophage therapy has potential as an innovative and effective therapy against *M*. *ulcerans* infection [9]. Indeed, our results in the murine model show that treatment with mycobacteriophage D29 decreases the proliferation of *M*. *ulcerans* in the subcutaneous tissue resulting in marked macroscopic improvement of skin lesions. Following this line of research, endolysins (lysins) are phage encoded enzymes produced during the late phase of the bacteriophage infection cycle, so as to degrade the cell wall peptidoglycan of the bacterial host, enabling the release of viral progeny [10–12]. Over the past decade, the development, characterization and exogenous application of recombinant and purified bacteriophage lytic enzymes has been successfully evaluated in several animal models of human diseases, such as sepsis, endocarditis, pharyngitis, pneumonia, meningitis and mucosal and skin infection [13–21]. Moreover, the use of a commercial endolysin for the treatment of *Staphylococcus aureus* skin infections has already been approved [22].

Mycobacteria have an unusual outer membrane composed by mycolic acids esterified with arabinogalactan (AG), which is linked to peptidoglycan, forming the mycolylarabinogalactan-peptidoglycan (mAGP) complex, a potential barrier to phage-mediated lysis [23]. Mycobacteriophage genome sequences show that, in addition to lysins that degrade the peptidoglycan layer of bacterial cell walls [24–26], some mycobacteriophages also encode a second lysin that targets the mAGP complex, known as Lysin B (LysB) [27–29]. As described by Payne *et al*. [27], mycobacteriophage D29 encodes LysB, a mycolylarabinogalactan esterase that cleaves the ester linkage joining the mycolic acid-rich outer membrane to AG, releasing free mycolic acids [30]. Although few studies have shown that LysB can also act externally, suggesting its promising antimicrobial effect, there is no proven efficacy *in vivo* [31].

In the present study, following the *in vitro* evaluation of the lytic activity of recombinant mycobacteriophage D29 LysB against *M*. *ulcerans* isolates, the therapeutic effect of LysB during *M*. *ulcerans* infection was evaluated in the mouse footpad model of infection. The progression of macroscopic/microscopic pathology and bacterial loads, as well as the cytokine profiles, were evaluated in the footpad and the draining lymph node (DLN).

## Materials and methods

### Bacteria and culture conditions

*Mycobacterium smegmatis* mc^2^, *Mycobacterium bovis* BCG and *Mycobacterium tuberculosis* H37Rv (PREMAS Biotech, New Delhi) were grown in Middlebrook 7H9 broth with or without ADC, at 37°C. *M*. *ulcerans* strains (Institute of Tropical Medicine, Antwerp) were grown on Middlebrook 7H9 supplemented with 1.5% agar at 32°C for approximately 6–8 weeks. Other bacteria used in this study, *S*. *aureus* ATCC 29213, *Bacillus subtilis* HER1243, *Enterococcus faecalis* ATCC 29213, *Escherichia coli* ATCC 25922, *Klebsiella pneumoniae* MTCC109, and *Pseudomonas aeruginosa*, were grown in Mueller-Hinton broth.

### Expression and purification of LysB

The mycobacteriophage D29 LysB gene (GenBank: accession number AF022214) was amplified by PCR from mycobacteriophage D29 DNA using the primer: 5´-CCCTGGAACATATGAGCAAGCCC-3´ (nt 6606 to 7370). The amplification product was cloned into the expression vector pET28a fused to an N-terminal 6xHis tag. The resulting plasmid pET28a–LysB was used to overexpress LysB using *E*. *coli* BL21 (DE3) as a host for expression. Expression cultures were grown to an optical density (OD) between 0.4 and 0.6 at 600 nm, in Luria-Bertani broth containing kanamycin (50 μg/mL). Protein expression was induced with 1mM isopropyl-D-thiogalactopyranoside (IPTG) with shaking for 4 h at 37°C. Bacterial cells were harvested by centrifugation (10000xg, 5 min, 4°C), resuspended in phosphate buffer (50 mM NaH_2_PO_4_, 300 mM NaCl, 10 mM imidazole, pH 8), sonicated on ice for 5x 10 s pulses separated by 10 s rests and then centrifuged (10000x g, 5 min, 4°C). For purification, the supernatant was applied to a nickel-nitrilotriacetic acid (Ni-NTA) agarose column and the protein was eluted under native conditions with 500 mM imidazole in phosphate buffer according to the manufacturer’s instructions. The purity of the protein was analyzed by 12% sodium dodecyl sulfate-polyacrylamide gel electrophoresis (SDS-PAGE) followed by Coomassie blue staining. Protein-containing fractions were combined and filter sterilized (0.22 μm). Protein concentration was determined using NanoDrop ND-1000. For more details, please see S1 Fig.

### Site directed mutagenesis

The S82A mutation in LysB encoding gene was introduced by site directed mutagenesis kit (Strategene) according to the protocol provided by the manufacturer. pGDC403 was used for introducing the mutation using the following primers: forward (GMB820) - 5’GATGGCGGGTTACGCGCAGGGAGCCAT-3’ and reverse (GMB821)- 5’GATGGCTCCCTGCGCGTAACCCGCCAT-3’. The PCR conditions were as follows: 95°C– 2 min, 95°C– 30 s, 60°C– 30 s, 66°C– 30 s. The amplified product was treated with DpnI for 2 h at 37°C followed by transformation into *E*. *coli* Top 10 cell. The mutation was confirmed by gene sequencing and the plasmid was designated as pGDC523.

### Calcium precipitation assay

The lipolytic activity of the purified LysB was confirmed by spotting on Middlebrook 7H9 plates containing substrates as follows: 1% (v/v) Tween 20 with 1 mM CaCl_2._Plates were incubated at 37°C for at least 24 h. Enzymatic activity was indicated by the formation of a white precipitate spot [28,32].

### PNP release assay

The release of p-Nitrophenol (PNP) by lipase activity of LysB on p-nitrophenol butyrate (PNPB) was measured by using 200 μl reaction mixture containing 50 μg of purified LysB and 10 mM PNPB in 25 mM Tris buffer (pH 7.2) at room temperature for 15 minutes followed by measuring at 410 nm.

### Drug susceptibility assay

The minimum inhibitory concentration (MIC) determination for *M*. *smegmatis*, *M*. *bovis* BCG, *M*. *tuberculosis*, *and M*. *ulcerans* was performed using the microtitre plate based colorimetric assay. LysB protein or drug of a known concentrations were double diluted serially in media in a 96-well plate from the 2^nd^ well to 11^th^ well. The 1^st^ well was used as media control and the 12^th^ as cell control. Bacterial suspensions were added to achieve 3–5 X 10^5^ CFU/ml in each well. The plates were incubated at 37°C for 3 days for *M*. *smegmatis*, 7 days for *M*. *bovis* BCG and *M*. *tuberculosis*, and 15 days for *M*. *ulcerans*. At the end of incubation period, resazurin dye (0.02%) with 10% Tween 80 was added to all the wells and the MIC was determined spectroscopically at 575 and 610 nm. The MIC was defined as the lowest concentration of the protein or drug showing 80% inhibition of growth.

For the MIC determination in Gram-positive and Gram-negative bacteria, microtitre plates were prepared as described above and incubated at 35°C for 16–18 hours. After the incubation period, plates were read spectrophotometrically at 600 nm.

### Plate lysis assay

The plate lysis assay was performed as previously described [33]. Clinical isolates were grown in Middlebrook 7H9 broth at 32°C to an OD_600_ of 1.0 and clumps were dispersed by passing the bacterial suspension several times through a 25-gauge needle. The bacterial suspension (10^5^ CFU/mL) was plated on Middlebrook 7H9 supplemented with 1.5% agar. A stock solution of purified LysB was serially diluted in phosphate buffer (final concentration 10–0.1 μg/mL) and spotted onto bacterial lawns that air dried for 30 min. Phosphate buffer was spotted as a negative control. Plates were incubated at 32°C for approximately 6–8 weeks. Antimicrobial activity of LysB was indicated by a clear lysis zone within the lawn where *M*. *ulcerans* growth was prevented.

### Checkerboard MIC assay

The combination MIC experiments were performed in a 96-well plate. In the first step, drug was double diluted row wise, while LysB was double diluted column wise. One column was maintained without drug and one row was maintained without protein to serve as controls. Bacterial cultures containing 3-5x10^5^ CFU/ml was added to the wells and incubated for 3 (*M*. *smegmatis*), 7 (*M*. *bovis* BCG or *M*. *tuberculosis*), or 15 days (*M*. *ulcerans*). Resazurin dye was added and MIC was determined as described above. The fractional inhibitory concentration (FIC) index was calculated as FIC of protein + FIC of drug. FIC of protein or drug was calculated as MIC of protein or drug in combination/MIC of drug or protein alone. FIC Index of < 0.5 was considered synergy, 0.5–1.0 additive, 1.0–4.0 indifferent and > 4.0 as antagonistic.

### Haemolysis assay

LysB was serially diluted in PBS and human red blood cells were added at 10% haematocrit. The 96-well plate was incubated at 37°C for 1 h, followed by centrifugation at 3000 rpm for 15 min. 100 μL supernatant was transferred to a fresh plate and the plate was read at 540 nm using Spectramax. PBS and Triton-X100 were included in the assay as negative and positive controls respectively. The percentage of haemolysis was calculated by considering positive control value as 100%.

### *In vivo* bioavailability and enzymatic activity of LysB

The bioavailability and lipolytic enzymatic activity of LysB were evaluated at different time points in sera, footpads and DLN of mice subcutaneously (s.c.) injected in the footpad with 50 μg of LysB. Sera were collected by retro-orbital bleeding, footpads were minced, resuspended in PBS and vortexed with glass beads, and DLN were passed through a 40 μM cell strainer. Footpad and DLN suspensions were further centrifuged for 10 min at 5000 rpm, and supernatant was considered the total protein extract. For the evaluation of protein bioavailability in samples, a western blot was performed. Briefly, samples were loaded and resolved by a 12% SDS-PAGE and transferred to 0.2 μm Nitrocellulose membranes (Bio-Rad) with the semi-dry Trans-Blot Turbo system (Bio-Rad). Membranes were blocked and subjected to immunoblotting with anti-mouse 6xHis antibody peroxidase conjugate (Clontech Lab. Inc., Takara). The 6xHis-tagged LysB was detected with SuperSignal (Thermo Scientific #34095) in a Universal Hood II (Bio-Rad). The enzymatic activity of LysB in samples was assessed by a lipase assay, as described above.

### Animals

Eight-week-old female BALB/c mice were obtained from Charles River (Barcelona, Spain) and were housed under biosafety level 3 conditions with food and water *ad libitum*.

### Footpad mouse model of *M*. *ulcerans* infection

For the preparation of inoculum, *M*. *ulcerans* 98–912 was recovered, diluted in PBS and vortexed using glass beads. Mice were s.c. infected in the left hind footpad with 0.03 ml of *M*. *ulcerans* suspension containing 5.5 log_10_ CFU.

### Treatment of *M*. *ulcerans*-infected mice with LysB

Treatment was initiated when footpads of mice were swollen to 2.7 mm and was performed by two s.c. injections in the infected footpad with 50 μg of LysB in PBS at 10- and 13-days post-infection. Control-infected mice were injected with PBS without protein. Two groups of uninfected animals were also injected with LysB or vehicle PBS buffer alone, as controls.

### Assessment of footpad swelling

After infection, as an index of lesion development, footpad swelling of mice was monitored throughout the experiment, using a caliper to measure the diameter of the frontal area of the footpad. For ethical reasons, mice were sacrificed after the emergence of ulceration and no further parameters were evaluated.

### Bacterial growth

*M*. *ulcerans* growth was evaluated in footpad tissues. Briefly, footpad tissue specimens were minced, resuspended in PBS and vortexed with glass beads to obtain homogenized suspensions. Serial dilutions of the footpad were plated on Middlebrook 7H9 supplemented with 1.5% agar. *M*. *ulcerans* numbers were counted after 6 to 8 weeks of incubation at 32°C and expressed as colony forming units (CFU).

### Detection of cytokines

The levels of the cytokines tumor necrosis factor (TNF) and gamma interferon (IFN-γ) in the supernatant of homogenized suspensions from DLN of mice were quantified by using a Quantikine Murine ELISA kit (R&D systems), according to the manufacturer’s instructions.

### Ethics statement

This study was approved by the Portuguese national authority for animal experimentation *Direção Geral de Alimentação e Veterinária* (DGAV 8421 from 2018). Animals were kept and handled in accordance with the Directive 2010/63/EU of the European Parliament and of the Council, on the protection of animals used for scientific purposes (transposed to Portuguese law–*Decreto-Lei* 2013/113, 7th of august).

### Statistical analysis

Differences between the means of experimental groups were analyzed with the two-tailed Student t test. Differences with a P value of ≤ 0.05 were considered significant.

## Results

### Recombinant LysB is a specific inhibitor of mycobacteria

**Fig 1:**
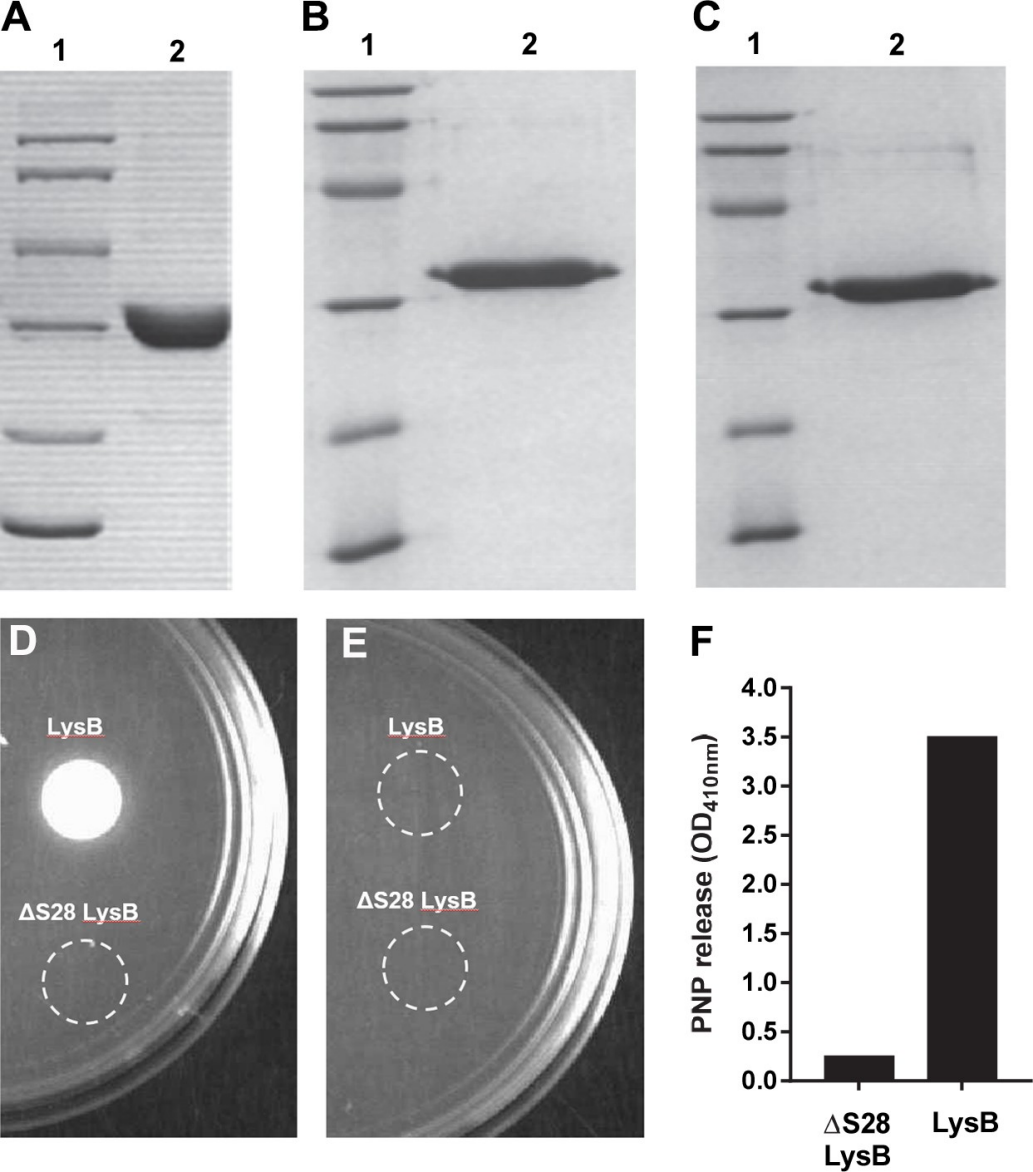
Lipolytic activity of LysB proteins. **(A)** Untagged, **(B)** His_6_-tagged protein and **(C)** 82A mutant LysB proteins were purified to > 90% homogeneity. Lane 1- molecular weight marker, lane 2–10μg protein. **(D-E)** The lipolytic activity of wild-type and mutant ΔS28 LysB was determined by the calcium precipitation assay using medium supplemented with 1 mM CaCl_2_ in the **(D)** presence or **(E)** absence of Tween-20 as substrate_._ Enzymatic activity is indicated by the formation of a white precipitate spot. **(F)** Lipase activity of wild type and mutant LysB proteins was also determined by the PNP release assay.

Mycobacteriophage D29 LysB was purified to > 90% homogeneity as untagged (Fig 1A) or His_6_-tagged protein (Fig 1B) and both proteins showed comparable biochemical activity. LysB lipolytic activity was tested using the CaCl_2_ precipitation assay on Middlebrook 7H9 agar plates containing Tween 20 as substrate [28,32]. Tween are esters of oleic (C18) and lauric (C12) acids, respectively, and can be cleaved by lipolytic enzymes to produce fatty acids and alcohol. The presence of Ca^2+^ in the medium causes the formation of an insoluble fatty acid salt that presents itself as a white precipitate. As observed in Fig 1D, LysB produced a spot of white precipitate, confirming the enzymatic lipolytic activity of purified recombinant mycobacteriophage D29 LysB. No enzymatic activity was observed with plates with no Tween substrate (Fig 1E).

In order to demonstrate that LysB-mediated activity is enzymatic in nature, the active site serine (S82) residue was changed to alanine. S82 had been earlier predicted to be a part of catalytic triad (S82, D166, H240) of LysB [27]. The intended mutation was confirmed by DNA sequencing of *lysB* gene. The mutant protein was purified (Fig 1C) and tested for enzymatic activity by CaCl_2_ precipitation assay and by para-nitrophenyl butyrate (PNPB). The mutant protein did not show any precipitation of CaCl_2_ in the presence of Tween 20 (Fig 1D) nor did it show any PNP release using PNPB as substrate, when compared to wild-type LysB (Fig 1F). This demonstrated that the alteration of S82 to A82 abolished the enzymatic activity of LysB protein.

LysB susceptibility assays were performed on different mycobacterial species, including *M*. *smegmatis*, *M*. *bovis* BCG and *M*. *tuberculosis*, and also on a number of Gram-positive and Gram-negative bacteria, such as *S*. *aureus*, *B*. *subtilis*, *E*. *faecalis*, *E*. *coli*, *K*. *pneumoniae* and *P*. *aeruginosa*. The MIC of LysB for *M*. *smegmatis*, *M*. *bovis* BCG and *M*. *tuberculosis* was found to be 1.5, 0.19 and 0.20 μg/ml, respectively, while LysB purified from ΔS28 lysB did not inhibit mycobacterial growth at the highest concentration tested (100 μg/ml), proving that enzymatic killing of mycobacteria was mediated through the active site serine residue. Regarding the antimicrobial activity of LysB against Gram-positive and Gram-negative bacteria, no inhibition was observed on bacterial proliferation, even at the highest concentration tested (500 μg/ml). Collectively, these results show that LysB is a specific inhibitor of mycobacteria.

**Fig 2:**
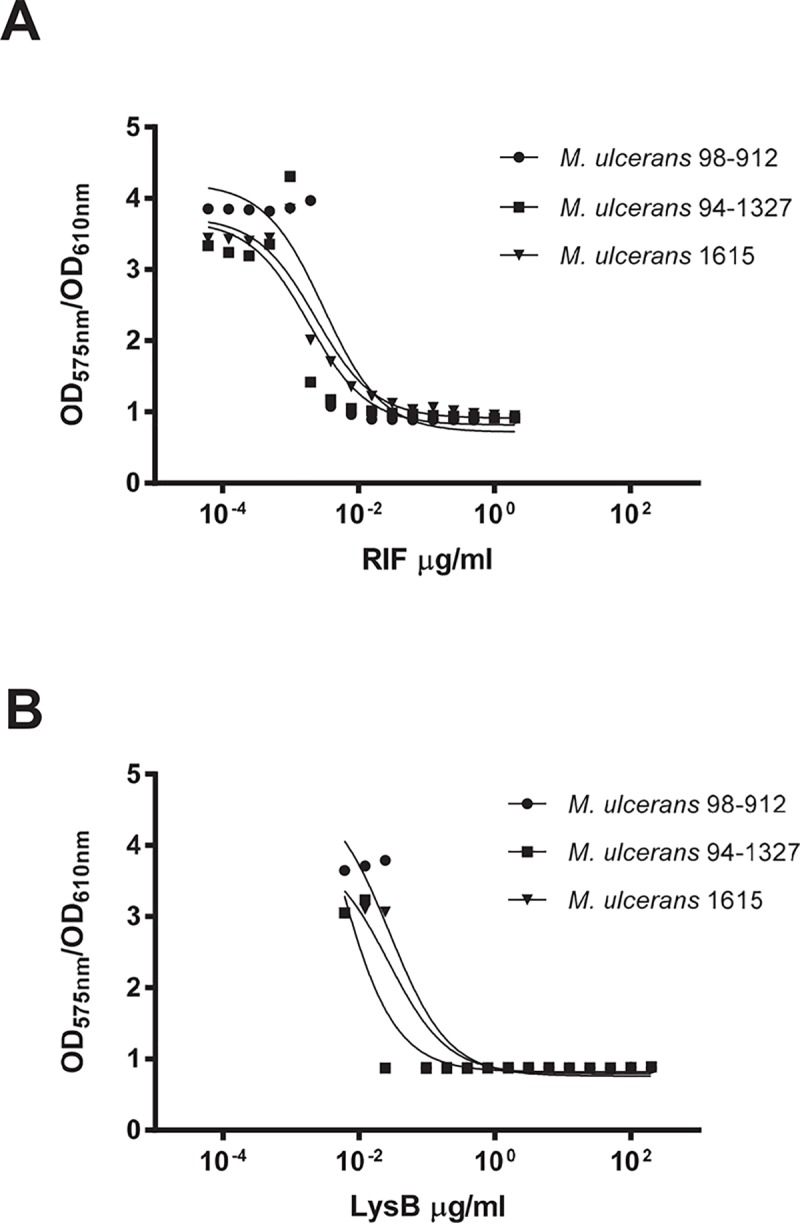
Antimicrobial activity of Rifampicin and LysB again*st M*. *ulcerans* isolates. The inhibitory effect of **(A)** RIF and **(B)** LysB against *M*. *ulcerans* strain 98–912 (circle), strain 94–1327 (square), and strain 1615 (triangle) were determined using the microtitre plate based colorimetric assay. Plots are representative of two independent experiments.

In that sense, we proceeded with the MIC determination for three representative strains of *M*. *ulcerans* that produce distinct types of mycolactone [34]: strain 1615, mycolactone A/B; strain 94–1327, mycolactone C; and strain 98–912, mycolactone D. The tested isolates showed susceptibility not only to the gold-standard rifampicin (Fig 2A), with a MIC of 0.0096 μg/ml, but also to LysB (Fig 2B), with MIC of 0.079 μg/ml. To further expand these results, purified LysB was tested against an extended panel of *M*. *ulcerans* isolates using the plate lysis assay. Representative isolates from endemic BU areas were selected based on their genetic and phenotypic characteristics, including the type of mycolactone produced and their virulence for mice (Table 1) [4,35–39]. Similar to the drug susceptibility assay, all *M*. *ulcerans* isolates tested were susceptible to the action of LysB protein, causing a clear spot zone indicating cell lysis in *M*. *ulcerans* lawns (Table 1).

**Table 1:** Antimicrobial activity of LysB again*st M*. *ulcerans* isolates.

*M*. *ulcerans* strain	Origin	Geographical origin	Type of Mycolactone	Lysin B (μg/mL) *
97–1116	Plaque	Benin	A/B	0.5
94–1331	nd	Papua New Guinea	A/B	0.5
5114	Ulcer	Mexico	-	0.1
00–1441	Aquatic insect	Benin	A/B	0.1
94–1324	Ulcer	Australia	C	0.1

* Minimum concentration of LysB tested causing a lysis zone in *M*. *ulcerans* lawns. The results are representative of three independent assays.

nd: not determined

### Combinations of LysB and antimycobacterial drugs are synergistic

In order to determine if LysB could show synergistic inhibitory effect with other antimycobacterial drugs, checkerboard assays were set up with LysB in combination with RIF using *M*. *smegmatis*, *M*. *bovis* BCG and *M*. *ulcerans* cultures. With *M*. *smegmatis*, we observed that LysB could lower the MIC value of RIF and the FIC index was calculated to be 0.06. The low FIC index (< 0.5) suggested that a combination of LysB and this anti-mycobacterial drug acts in a synergistic manner in inhibiting *M*. *smegmatis* growth. In the case of *M*. *bovis* BCG and *M*. *ulcerans*, the corresponding FIC index value was 0.56 and 1.0, respectively, when LysB was used in combination with RIF, suggesting an additive effect.

### LysB is enzymatically active *in vivo*

**Fig 3:**
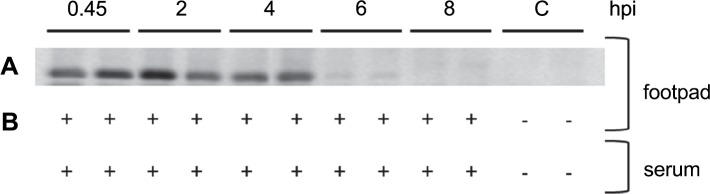
Assessment of bioavailability and enzymatic activity of LysB in the footpad and serum of mice. Mice were injected subcutaneously in the left footpad with LysB. At different time points, the presence of LysB was assessed by Western blot **(A)** and LysB enzymatic activity was determined by a lipase assay **(B)**, in footpad supernatant and serum; hpi, hours post-injection; C, control mice; + lipase activity;—no activity. Results are from one representative experiment of two independent experiments.

To determine the bioavailability and presence of enzymatically active LysB *in vivo* after s.c injection in the footpad, the protein was measured in footpads, DLN, and sera. In a western blot analysis of footpad supernatant, LysB was consistently detected, and the levels remained present during the first 4h after administration (Fig 3A). At 6 h after injection, LysB was still detected, although at lower levels (Fig 3A). In order to analyze if LysB maintained its lipolytic enzymatic activity *in vivo*, a lipase assay was performed [28,33]. As shown in Fig 3B, lipolytic activity was detected in footpad suspensions until 8h after injection. Regarding sera samples, although LysB was not detected by western blot analysis, lipolytic activity was observed (Fig 3B). No lipolytic activity or presence of LysB was detected in the DLN.

### Treatment with LysB prevents *M*. *ulcerans* proliferation in the footpad

**Fig 4:**
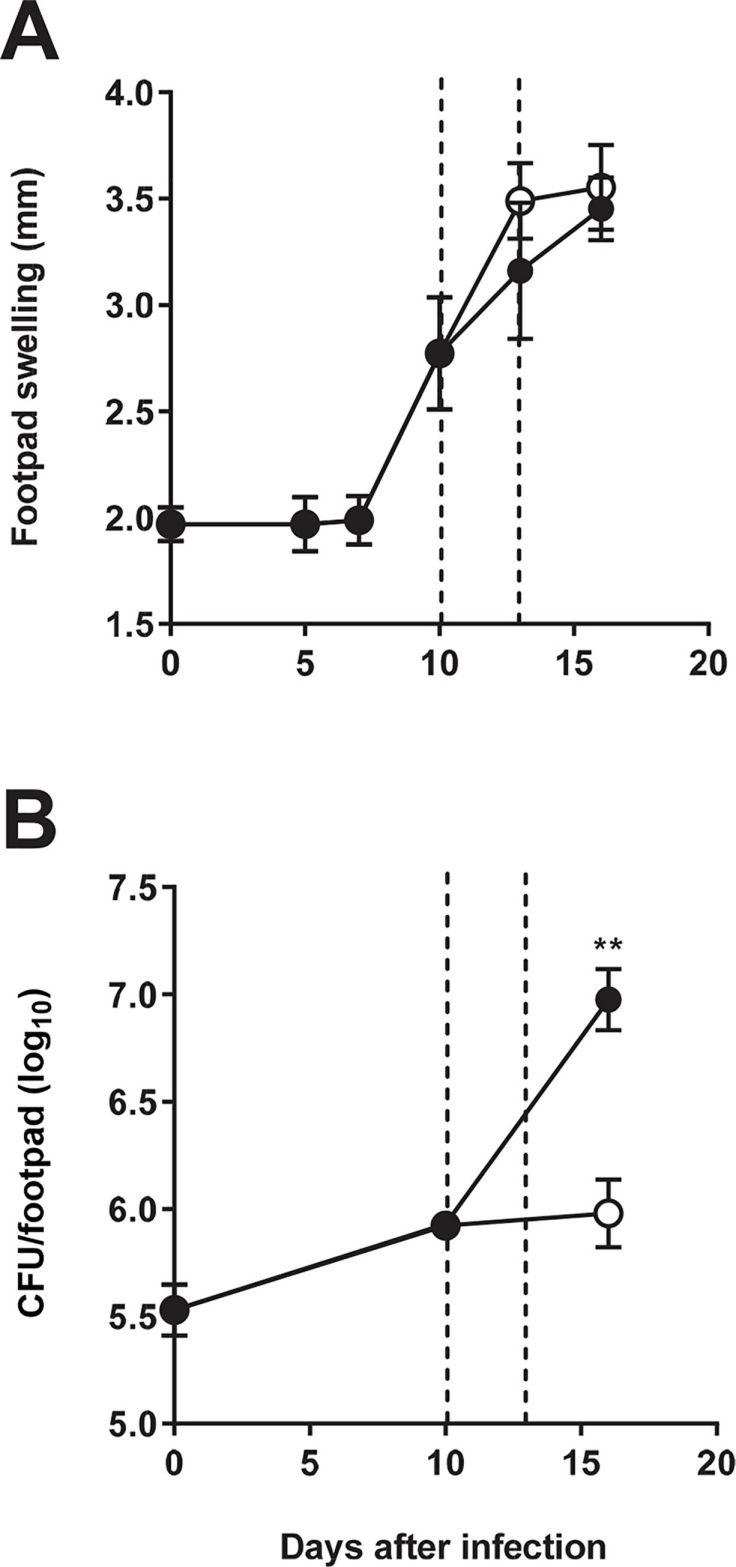
Lesion progression and *M*. *ulcerans* proliferation in footpads of infected mice. Mice were infected subcutaneously in the left footpad with 5.5 log_10_ CFU of *M*. *ulcerans* strain 98–912. After the emergence of macroscopic lesion (footpad swelling of 2.7mm) mice were subjected to treatment with two doses of subcutaneous injection of LysB (10 and 13 days post-infection—dashed lines). **(A)** Footpad swelling (n = 15) and **(B)** bacterial proliferation (n = 6) was assessed in non-treated *M*. *ulcerans* infected mice (black circles) and LysB treated *M*. *ulcerans* infected mice (white circles). Mice were sacrificed for ethical reasons after the emergence of ulceration. Results are from one representative experiment of two independent experiments. Data points represent the mean ± SD. Significant differences between treated and non-treated mice were performed using Student’s t test (**, p≤0.01).

To investigate the efficacy of LysB treatment for the control of *M*. *ulcerans in vivo*, we used the footpad mouse model of infection [4,9,36–39]. Mice were s.c. infected in footpads with 5.5 log_10_ CFU of *M*. *ulcerans* strain 98–912. After 10 days, when footpad swelling reached 2.7 mm (Fig 4A), and at 13 days post-infection, mice were treated subcutaneously in the footpad with 50 μg of LysB in PBS. As shown in Fig 4B, *M*. *ulcerans* proliferated in infected footpads of non-treated mice over the course of experimental infection (P<0.01), while Lys B treatment prevented further bacterial multiplication, resulting in a significant 1 log difference from untreated controls at day 16 post-infection. The administration of vehicle PBS or LysB alone in uninfected footpads did not induce significant swelling of the footpad. Collectively, these results show that the administration of LysB to *M*. *ulcerans* infected tissue has a protective effect, significantly preventing bacterial proliferation.

### Treatment with LysB induces increased levels of IFN-γ and TNF in the DLN

Previous studies from our laboratory showed that *M*. *ulcerans* disseminates to the DLN, where the differentiation/expansion of mycobacteria-specific specific T cells occurs, contributing for the control of *M*. *ulcerans* proliferation through the production of IFN-γ [36,39] and TNF [4]. To determine whether LysB treatment impacts host immune response, we carried out a comparative analysis of cytokine kinetics in the DLN.

**Fig 5:**
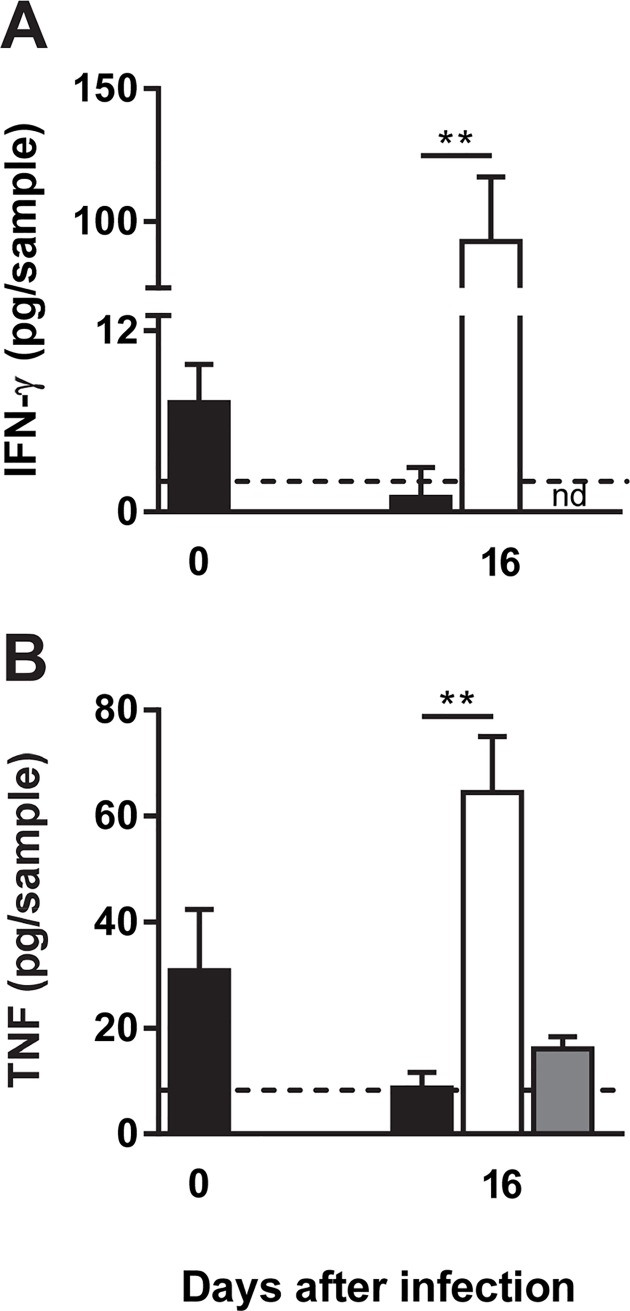
Cytokine profile in DLN of LysB-treated *M*. *ulcerans* infected mice. Mice were infected subcutaneously in the left footpad with 5.5 log_10_ CFU of *M*. *ulcerans* strain 98–912. After the emergence of macroscopic lesion (footpad swelling of 2.7mm) mice were subjected to treatment with two doses of subcutaneous injection of LysB (10 and 13 days post-infection). **(A)** Levels of IFN-γ and **(B)** TNF were quantified by ELISA in DLN of non-treated *M*. *ulcerans* infected mice (black bars), LysB treated *M*. *ulcerans* infected mice (white bars) and LysB treated non-infected mice (grey bars). Results are from one representative experiment of two independent experiments. Bars represent the mean ± SD (n = 6). n.d., not detected. Dashed lines represent the detection limit. Significant differences between treated and non-treated mice were performed using Student’s t test (**, p≤0.01).

Treatment with LysB resulted in a significant increase in the levels of IFN-γ in the DLN (P<0.01) at day 16 post-infection (six days after the beginning of the treatment), as compared with non-treated mice (Fig 5A). The protein levels of the pro-inflammatory cytokine TNF were low in the DLN of non-treated mice (Fig 5B). In contrast, in LysB-treated mice, significant levels of TNF (P<0.01) were detectable at day 16 post-infection (day 6 post-treatment) (Fig 5B).

## Discussion

We have previously shown in the mouse footpad model of *M*. *ulcerans* infection that a single s.c. injection of the lytic mycobacteriophage D29 can effectively decrease the proliferation of *M*. *ulcerans* resulting in marked macroscopic improvement of skin lesions [9]. However, the safe and controlled use of phages in humans still requires further experimentation to fulfill the scientific requirements of current pharmaceutical agencies. Therefore, there is currently a movement in favor of bacteriophage endolysins (lysins), as an alternative innovative therapeutic strategy. The potential of purified recombinant bacteriophage lytic enzymes (lysins) has been regarded as a viable method to control bacterial pathogens, including *S*. *aureus* [20,21,33,40,41], *Streptococcus pneumoniae* [13–15,17,19], group B streptococci [21], and *Bacillus anthracis* [42]. Recently, a new engineered endolysin (Artilysin Art-175) has shown activity against gram-negative pathogens [43]. In addition, endolysins have advantageous characteristics that avoid most of the common resistance mechanisms against antibiotics [42]. The potent anti-mycobacterial activity shown herein by LysB compares well with the antibacterial potency of lysins. Although the ability of lysins as antibacterial agents is due to their action as murein hydrolases that cleave peptidoglycan bonds of bacteria without an outer membrane or surface lipids and waxes, LysB specifically acts upon mycobacteria due to its activity as a mycolylarabinogalactan esterase that hydrolyses the ester linkage that joins the mycolic acid-rich outer membrane to arabinogalactan [27]. Thus, lysins and LysB, despite working by different mechanisms of action, show potent antibacterial activity which is most likely dur to their ability to mechanically weaken and disrupt the bacterial cell wall. This hypothesis is supported by the loss of biochemical and antimycobacterial activity in the S82A mutant and by the fact that LysB shows synergy with an antimycobacterial drug, which probably results from increased cell wall permeability in the presence of LysB.

Due to the importance of the mAGP-complex for the stability of the mycobacterial cell envelope [44], we were prompted to test the therapeutic potential of LysB in the context of *M*. *ulcerans* infection. In this study, we have demonstrated the potential of mycobacteriophage D29 LysB therapy against *M*. *ulcerans* infection in the mouse footpad model. Indeed, we show that treatment with LysB can effectively prevent proliferation of the highly virulent mycolactone-producing *M*. *ulcerans* strain 98–912, even at an advanced stage of *M*. *ulcerans* infection. Importantly, our *in vitro* results show a high susceptibility of several *M*. *ulcerans* isolates to LysB, indicating that its activity *in vivo* is not limited to *M*. *ulcerans* 98–912.

The therapeutic efficacy of LysB treatment depends on the presence of biologically active LysB *in vivo*. As previously described, a rapid decrease in lysin levels after administration can result in the decrease of the amount of active lysin [19,45]. Indeed, in a pneumococcal meningitis mouse model, the bacterial load in the cerebrospinal fluid increased as the concentration of Cpl-1 lysin decreased over time [19]. Based on these observations, and in order to improve the LysB bioavailability in mouse footpads, we decided to perform two s.c. administrations in the footpad. The subcutaneous route of administration was chosen because it allows almost complete absorption at the site of injection and therefore is considered an accurate measure of the amount of LysB necessary to be effective. Nevertheless, the possibility of developing a formulation for the topical application of lysins would be of great interest to increase patient compliance. Additionally, repeated administrations of LysB can be done to treat bacterial infections without adverse effects or loss of efficacy, since it has been demonstrated using different lysins and pathogens (*S*. *pneumoniae*, *S*. *aureus*, *Streptococcus pyogens* and *B*. *anthracis*) [46], that the development of antibodies against lysins are non-neutralizing. In fact, when naïve and lysin-immunized mice were challenged with *S*. *pneumoniae* and afterwards treated with Cpl-1 lysin, no differences were observed between the groups of mice regarding reduction of bacterial numbers [45].

It is known that the differentiation/proliferation of IFN-γ producing mycobacteria-specific lymphocytes occur in mouse DLN early after experimental *M*. *ulcerans* infection [36]. However, this transient protective host response is not sufficient to inhibit the proliferation of virulent *M*. *ulcerans* in mice, as increasing concentrations of mycolactone at the infection site impair the effector activity of macrophages and induce cell and tissue destruction [4,38,39]. In our study, LysB administration effectively decreased the bacterial load in the infected tissue, which likely resulted in a reduction of mycolactone levels and allowed the continuous development of a protective host immune response and a significant increase in the levels of pro-inflammatory cytokines in the DLN.

For therapeutic use, an antimicrobial agent should not affect mammalian cells and only targeting the pathogen. Lysins target structures unique and highly conserved to bacterial cells and as such should not present a potential toxic threat to humans and animals [47]. This has been supported by several reports using lysins in preclinical studies in mouse models [15,45,48] and, in agreement, in our study, since the administration of LysB did not result in increased haemolysis *in vitro* and was not associated with detectable side effects in the mouse model until the end of the experimental period.

This is the first study on mycobacteriophage LysB activity against *M*. *ulcerans* infection. Importantly, LysB could be used as an adjuvant to improve the current antibiotic regimen, given its *in vitro* additive effect when in combination with rifampicin. Although the development of a treatment protocol using LysB will require further optimization, namely regarding the optimal mode of administration, dosage and schedule, this is the first study providing proof of concept of the antimicrobial activity of LysB against *M*. *ulcerans* infection.

## Supporting information

S1 Fig Purification of various LysB proteins. Purification of His tagged native (A) and 82A mutant of LysB (B) was done by Ni affinity chromatography, while the untagged native LysB was purified by conventional chromatography (C). In A and B, lane 1 –cell pellet, lane 2 –cytosolic fraction (load), lane 3 –molecular size marker, lane 4 –flow through, lane 5 –imidazole wash, lane 6 and 7–10 and 20 μl of the eluate fractions. C shows ion exchange chromatography purification. Lane 1 –load, lane 2 –molecular size marker, lane 3 and 4 –flow through, lane 5 and 6 –wash fractions, lanes 7 to 16 –eluates.
(DOCX)
