# Supplementary material for: Antimicrobial activity of Mycobacteriophage D29 Lysin B during Mycobacterium ulcerans infection
Source: PLoS Negl Trop Dis. 2019 Aug 19;13(8):e0007113. doi: 10.1371/journal.pntd.0007113 (PMC6730932; doi:10.1371/journal.pntd.0007113)
Supplement: S1 Fig — Purification of His tagged native (A) and 82A mutant of LysB (B) was done by Ni affinity chromatography, while the untagged native LysB was purified by conventional chromatography (C). In A and B, lane 1 –cell pellet, lane 2 –cytosolic fraction (load), lane 3 –molecular size marker, lane 4 –flow through, lane 5 –imidazole wash, lane 6 and 7–10 and 20 μl of the eluate fractions. C shows ion exchange chromatography purification. Lane 1 –load, lane 2 –molecular size marker, lane 3 and 4 –flow through, lane 5 and 6 –wash fractions, lanes 7 to 16 –eluates. (DOCX) [file pntd.0007113.s001.docx]

**Supporting Information**

**Methods**

**Plasmid constructs**

The full-length gene encoding His tagged LysB was amplified from phage D29 DNA using the following primers: forward (GMB763) - 5’GCCGAATTCCATATGCATCATCATCATCATCATAGCAAGCCCTGG CTGTTCA 3’ and the reverse (GMB764)- 5’GCCGAATTCAAGCTTTCAGATCTGTCGTAGGAACTCGAC 3’. The PCR amplification conditions were 95^o^C – 5 mins, 95^o^C – 30 Sec, 60^o^C – 30 sec, 72^o^C – 1 min, The PCR was carried out using 30 cycles followed by a final 10 min extension at 72^o^C. The PCR product was cloned into NdeI-HindIII sites of pET26B to obtain pGDC403. The untagged LysB encoding gene was cloned from phage D29 by amplification using the following primers: forward (GMB1012)- 5’GCCGAATTCCATATGAGCAAGCCCTGGCTGTTCA 3’ and the reverse (GMB764) - 5’GCCGAATTCAAGCTTTCAGATCTGTCGTAGGAACTCGAC3’. the PCR amplification conditions were same as for the gene encoding His tagged LysB described above. The PCR product was cloned into NdeI-HindIII sites of pET26B to obtain pGDC523.

**Protein purification**

The WT (P403) and the mutant His tagged (P405) LysB were purified by Ni affinity chromatography (Fig S1). Briefly, the protein expressing *E. coli* /ER2566 (T7 expression host) cells were induced with IPTG and grown till OD_600_ = 7.4. The cell pellet was resuspended in 25 mM Tris buffer (pH 7.5) and lysed by sonication. The sonicated cells were centrifuged for 30 min at 10,160 g at 4°C. The clear supernatant was taken for loading onto 8.5 ml of Ni NTA resin pre-equilibrated with 25 mM Tris buffer (pH 7.5). Following this the column was washed with 3 column volumes of equilibration buffer followed by 3 column volumes of 25 mM Tris buffer (pH 7.5) containing 100 mM imidazole. The bound protein was eluted using 1M imidazole in 25 mM Tris buffer (pH 7.5). The eluted protein was dialyzed against 25 mM Tris buffer (pH 7.5). The dialyzed protein (2.3 mg/ml) was filter sterilized using a 0.2 µM filter before using for activity assays.

**Supporting Figure**


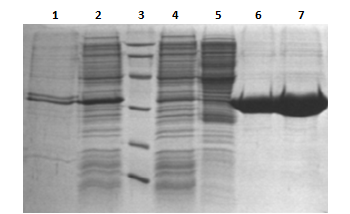

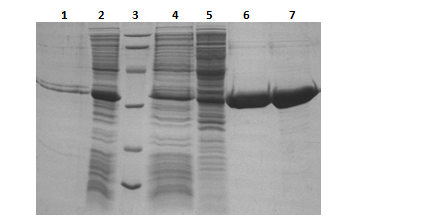

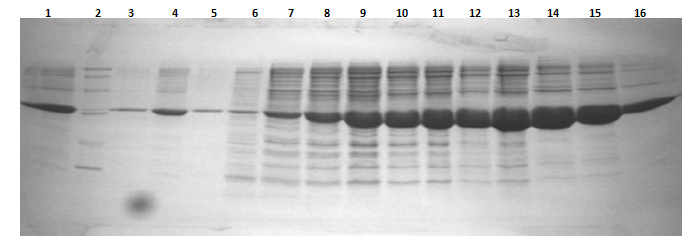


**A**

**B**

**C**

**S1 Figure**. Purification of various LysB proteins. Purification of His tagged native (A) and 82A mutant of LysB (B) was done by Ni affinity chromatography, while the untagged native LysB was purified by conventional chromatography (C). In A and B, lane 1 – cell pellet, lane 2 – cytosolic fraction (load), lane 3 – molecular size marker, lane 4 – flow through, lane 5 – imidazole wash, lane 6 and 7 – 10 and 20 µl of the eluate fractions. C shows ion exchange chromatography purification. Lane 1 – load, lane 2 – molecular size marker, lane 3 and 4 – flow through, lane 5 and 6 – wash fractions, lanes 7 to 16 – eluates.
